# Redox responsive activity regulation in exceptionally stable supramolecular assembly and co-assembly of a protein†

**DOI:** 10.1039/d0sc05312k

**Published:** 2020-11-17

**Authors:** Saptarshi Chakraborty, Rajesh Khamrui, Suhrit Ghosh

**Affiliations:** School of Applied and Interdisciplinary Sciences, Indian Association for the Cultivation of Science 2A and 2B Raja S. C. Mullick Road Kolkata India-700032 psusg2@iacs.res.in

## Abstract

Supramolecular assembly of biomolecules/macromolecules stems from the desire to mimic complex biological structures and functions of living organisms. While DNA nanotechnology is already in an advanced stage, protein assembly is still in its infancy as it is a significantly difficult task due to their large molecular weight, conformational complexity and structural instability towards variation in temperature, pH or ionic strength. This article reports highly stable redox-responsive supramolecular assembly of a protein Bovine serum albumin (BSA) which is functionalized with a supramolecular structure directing unit (SSDU). The SSDU consists of a benzamide functionalized naphthalene-diimide (NDI) chromophore which is attached with the protein by a bio-reducible disulfide linker. The SSDU attached protein (NDI-BSA) exhibits spontaneous supramolecular assembly in water by off-set π-stacking among the NDI chromophores, leading to the formation of spherical nanoparticles (diameter: 150–200 nm). The same SSDU when connected with a small hydrophilic wedge (NDI-1) instead of the large globular protein, exhibits a different π-stacking mode with relatively less longitudinal displacement which results in a fibrillar network and hydrogelation. Supramolecular co-assembly of NDI-BSA and NDI-1 (3 : 7) produces similar π-stacking and an entangled 1D morphology. Both the spherical assembly of NDI-BSA or the fibrillar co-assembly of NDI-BSA + NDI-1 (3 : 7) provide sufficient thermal stability to the protein as its thermal denaturation could be completely surpassed while the secondary structure remained intact. However, the esterase like activity of the protein reduced significantly as a result of such supramolecular assembly indicating limited access by the substrate to the active site of the enzyme located in the confined environment. In the presence of glutathione (GSH), a biologically important tri-peptide, due to the cleavage of the disulfide bond, the protein became free and was released, resulting in fully regaining its enzymatic activity. Such supramolecular assembly provided excellent protection to the protein against enzymatic hydrolysis as the relative hydrolysis was estimated to be <30% for the co-assembled protein with respect to the free protein under identical conditions. Similar to bioactivity, the enzymatic hydrolysis also became prominent after GSH-treatment, confirming that the lack of hydrolysis in the supramolecularly assembled state is indeed related to the confinement of the protein in the nanostructure assembly.

## Abstract 2

Supramolecular structure directing unit regulated co-assembly of a protein produces a highly stable fibrillar nanostructure and glutathione responsive release of the protein in its active state.

## Graphical abstract

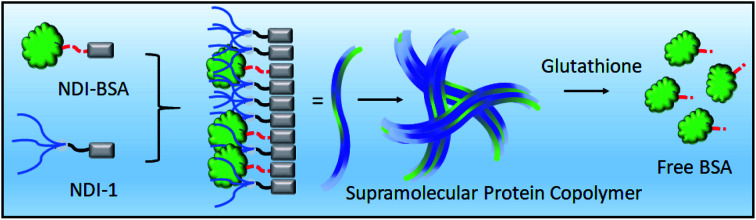

## Introduction

**Scheme 1:**
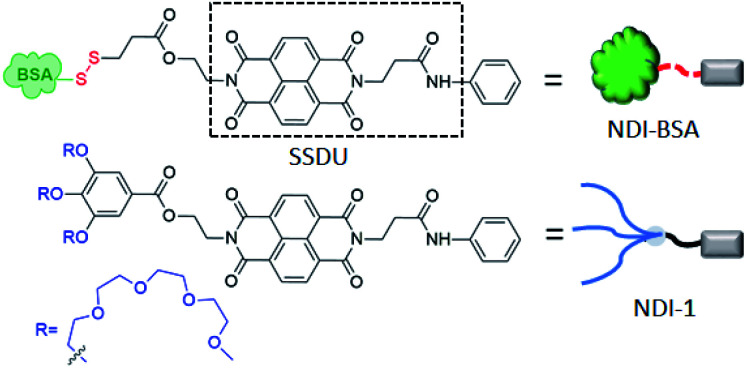
Structure of NDI-BSA and NDI-1.

Assembly of biomolecules is ubiquitous in nature and plays a vital role in important biological structures and functions.^1^ Prominent examples include formation of collagen networks, amyloid plaques, actin filaments or extremely sophisticated functions such as cell signalling, replication of the genetic code, gene translation and so on. Such self-assembly processes are governed by directional molecular interactions (H-bonding, π-stacking, metal–ligand coordination, ion–π interaction and others) amongst various functional groups present in the biomolecules or biomacromolecules. Inspired by their precision in structure and ability to perform many complex functions, researchers have sought to mimic them by suitable molecular engineering with different biological molecules and macromolecules for creating innovative synthetic biomaterials.^2^ Over the past few decades, assembly of molecularly engineered DNA,^3^ RNA,^4^ oligosaccharides,^5^ peptides^6^ or proteins^7^ have been studied with great interest by employing different non-covalent interactions. Despite significant progress in the field of DNA nanotechnology or peptide assembly, precision supramolecular assembly of proteins still remains a daunting task primarily because unlike DNA, proteins lack well-defined recognition sites for predictable and controllable supramolecular assembly. Furthermore, due to their large molecular weight, conformational complexity and structural instability as a function of temperature, pH or ionic strength, it remains to be a challenge to establish generally applicable strategies for protein assembly. However, protein assembly holds great promise for developing a fundamental understanding for various neurodegenerative diseases and also as promising functional materials. Protein based self-assembled materials have been explored in diverse applications such as antibacterial hybrid coating,^8^ regulation of sophisticated biomineralization processes,^9^ nanofabrication on surfaces,^10^ synthesis of free-standing 2D conducting metal films,^11^ guiding crystallization pathways,^12^ synthesis of nanofilms with encapsulation and release ability,^13^ molecular separation and dialysis,^14^ rapid synthesis of amyloid-based biomaterials^15^ biosensing,^16^ biocatalysis,^17^ and so on. In the recent past, protein-based therapy has emerged as a promising approach^18^ to treat various diseases such as cancers, diabetes, lysosomal storage disorders and others. In this context protein–polymer conjugates^19^ have been studied with great interest. However, despite growing interest in the field, poor cellular uptake and biodistribution, degradation of protein by enzymes (trypsin, pepsin, and α-chymotrypsin)^20^ and synthetic challenges associated with quantitative covalent conjugation of a protein with a polymer are major pitfalls which limit the scope for protein–polymer conjugates in biomedical applications.^20^ In most examples of protein–polymer conjugates, one polymer (generally PEG or other hydrophilic polymer) is attached with one protein with the objective to encase the protein surface by the polymer for protecting it from enzymatic degradation and achieving other advantages of PEGylation. However, intuitively this strategy may not produce the desired results because it is unlikely that protecting the surface of a protein will be effective by a single hydrophilic polymer, and the particle size of the conjugate in the absence of self-assembly still may not be large enough for avoiding rapid clearance. Therefore, it is not surprising that in the emerging literature of protein–polymer conjugates, there is hardly any example that actually reveals to what extent the enzymatic degradation of proteins could be prevented by polymer conjugation. In the recent past we have reported^21^ supramolecular structure-directing unit (SSDU) regulated entropy driven assembly of hydrophilic polymers by directional molecular interaction in which the self-assembly and morphology of the brush like supramolecular polymer^22^ could be regulated by specific molecular interactions instead of the packing parameter. We envisaged this could be equally effective for programmable assembly of SSDU-attached biomacromolecules such as proteins^23^ with the potential to address some of the pitfalls of protein–polymer conjugates as discussed before. With this objective, we have synthesized NDI-BSA (Scheme 1) in which a protein Bovine serum albumin (BSA) is attached with a SSDU that consists of a naphthalene-diimide (NDI) chromophore and an amide group. The design of the SSDU originates from our previous report in which we showed NDI-1 (Scheme 1), having a similar functionality, producing a linear supramolecular polymer in water by H-bonding and π-stacking.^24^ In this article we report the synthesis of NDI-BSA, its stimuli (temperature, redox) responsive self-assembly as well as co-assembly with NDI-1, impact of such supramolecular bioconjugation on thermal and hydrolytic stability of the protein and glutathione (GSH)-responsive release and retrieval of its enzymatic activity.

## Results and discussion

### Synthesis and characterization

**Scheme 2:**
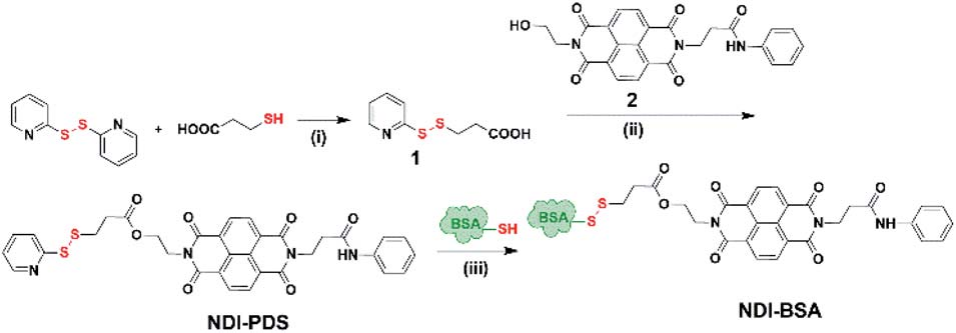
Synthesis of NDI-BSA. Reagents and conditions: (i) DCM/MeOH, CH_3_COOH, 5 h, 80%; (ii) DCM/DMF, EDC, 0–25 °C, 36 h, and 75%; (iii) 2.0 mM EDTA, PBS/DMF, 12 h, and rt.

**Fig. 1:**
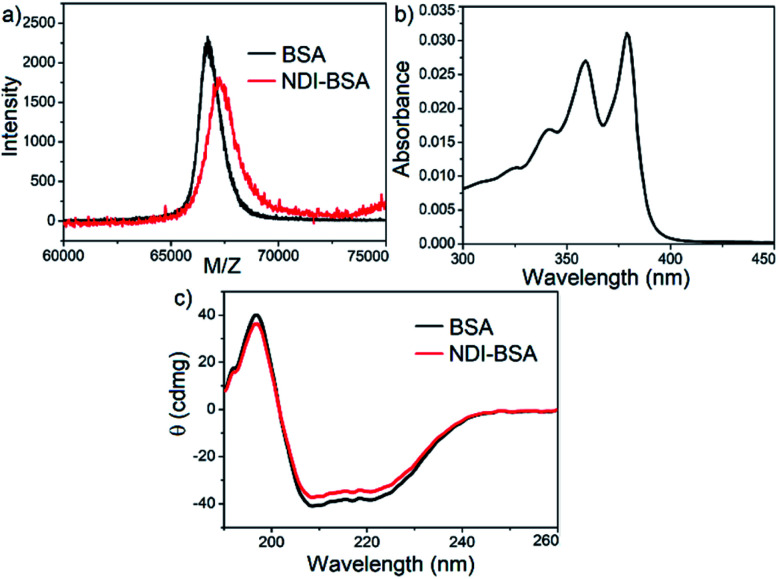
(a) MALDI-TOF MS spectra of BSA and NDI-BSA, matrix-sinapinic acid; (b) UV/Vis spectrum of GSH-treated NDI-BSA in DMF; (c) CD spectra of BSA and NDI-BSA in water (*c* = 0.05 mM, *l* = 0.1 cm).

Synthesis of NDI-BSA is depicted in Scheme 2. Benzamide functionalized NDI derivative 2 was coupled with compound 1 to produce NDI-PDS with the reactive pyridyl-disulfide group. Reduced BSA was prepared from commercially available BSA (see the ESI† for detail). Free thiol content of the reduced BSA was estimated to be 96% by the Ellman's assay (Fig. S1†). Comparison of the MALDI-TOF mass spectrometry data of the native and reduced BSA (Fig. S2†) confirmed absence of any residual protein-dimer peak after the reduction and lack of any other undesired conjugation during the reduction. The reduced BSA was treated with NDI-PDS to get the molecularly engineered protein NDI-BSA by thiol-disulfide exchange reaction and the resulting conjugate was isolated as a white powder. MALDI-TOF mass spectrometry^25^ of NDI-BSA revealed (Fig. 1a) a prominent peak with *m*/*z* = 67 245 and a clear shift was noticed in the peak position compared to that of the free BSA (*m*/*z* = 66 700). The difference of 545 mass units between the two peaks exactly matches with the mass of the attached NDI containing SSDU, confirming successful conjugation. Furthermore, the purity of NDI-BSA could be indirectly confirmed by estimating its molecular weight by UV/Vis spectroscopy (for details see the ESI†). NDI-BSA was found to be soluble only in water but not in other polar organic solvents like MeOH or DMF. But in water the UV/Vis spectra showed features of the aggregated NDI chromophore (*vide infra*). Therefore, the aqueous solution (known concentration in mg per mL unit) of NDI-BSA was treated with excess glutathione (GSH) for extended time to ensure complete cleavage of the disulfide bond and the mixture then was lyophilized, re-dispersed in measured volume of DMF, centrifuged and the clear supernatant was analysed by UV/Vis spectroscopy (Fig. 1b). It showed distinct absorption bands in the window of 300–400 nm corresponding to the monomeric NDI dye. In an independent experiment, the molar extinction coefficient of monomeric NDI-1 in DMF was estimated by concentration dependent UV/Vis studies (Fig. S3†). This value was used to estimate the concentration of the NDI chromophore in the DMF solution of the GSH-treated NDI-BSA from its absorption band intensity (Fig. 1b). Correlating the experimentally estimated concentration of the NDI chromophore and the concentration of NDI-BSA (in mg per mL unit), molecular weight of NDI-BSA was estimated to be 67 180 g per mole, which was remarkably close to the theoretically estimated value (66 430 + 545 = 66 975 g per mole) of NDI-BSA and thus indicated very high purity of the SSDU–protein conjugate. Far-UV circular dichroism (CD) spectrum of NDI-BSA (Fig. 1c) was identical to that of the native protein and it showed a negative band at 223 nm and a transition at relatively lower wavelength which was split into a negative band at 208 nm and a positive peak at 192 nm, indicating that the α-helix structure of BSA was undamaged in NDI-BSA.^26^

Biocompatibility of NDI-BSA was evaluated against HeLa cells by the MTT assay which revealed (Fig. S4†) the relative cell viability of about 90% after 48 h of incubation with NDI-BSA (*c* ≤ 500 μg mL^−1^). This was comparable to the native protein (Fig. S4†) and thus provided a preliminary indication that the conjugation with the NDI moiety did not reduce the biocompatibility of the protein.

### Supramolecular assembly

**Fig. 2:**
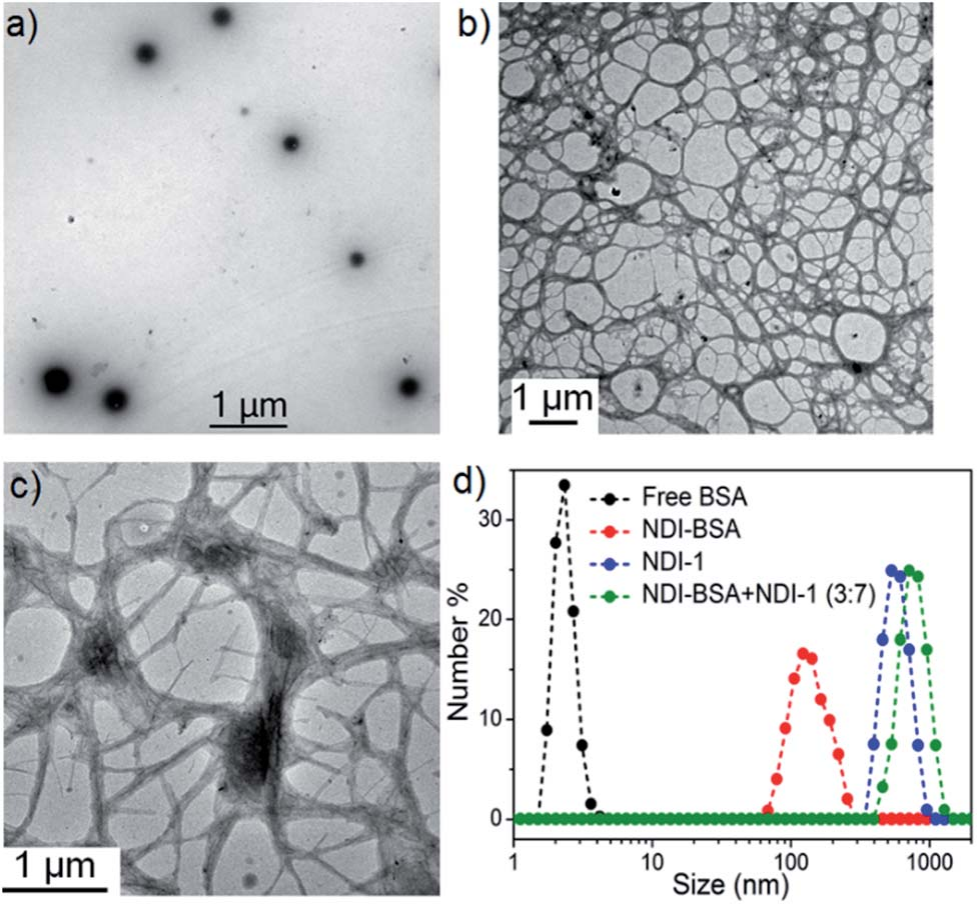
TEM images of (a) NDI-BSA, (b) NDI-1 and (c) NDI-BSA + NDI-1 (3 : 7); (d) number averaged size distribution of BSA, NDI-BSA, NDI-1 and NDI-BSA + NDI-1 (3 : 7) from DLS measurements (*c* = 0.05 mM in all samples). Autocorrelation function corresponding to the DLS plots are shown in the ESI (Fig. S6†).

We sought to examine the supramolecular assembly of NDI-BSA in an aqueous medium in which it produced a free-flowing solution. The transmission electron microscopy image of NDI-BSA shows the (Fig. 2a) formation of spherical nanoparticles with diameter in the range of 150–200 nm. In sharp contrast, an entangled 1D fibrillar (width 5–40 nm and some thicker fibrils) network was produced by the gelator NDI-1 (Fig. 2b). The presence of a few larger particles (Fig. 2a) or thicker fibrils (Fig. 2b) may be attributed to the drying effects and/or the inherent dispersity of the supramolecularly assembled structures. Although in both cases the SSDU is the same, such contrasting structures indicate a change in the supramolecular assembly pattern of the SSDU by conjugation with the large biomacromolecules. Interestingly, a mixed sample of NDI-BSA + NDI-1 (3 : 7) produced neither a free flowing solution like NDI-BSA nor gel like NDI-1, instead a highly viscous solution. A TEM image of the mixture revealed (Fig. 2c) the absence of any nanoparticle or thin fibre, but an entangled fibrillar network with width of the fibre in the range of 80–100 nm and length extending over few micrometres. Likewise, atomic force microscopy (AFM) images showed (Fig. S5†) nanoparticles (height ∼7 nm, width ∼150–200 nm) for NDI-BSA, and different looking fibrillar morphologies for NDI-1 and NDI-BSA + NDI-1 (3 : 7), similar to TEM images. Minor differences observed between the TEM and AFM images may be attributed to the different substrates and sample preparation methods in these two measurements. Dynamic light scattering (DLS) results (Fig. 2d) revealed a sharp peak for the native protein with hydrodynamic diameter (*D*_h_) <5.0 nm which is consistent with previous reports for globular proteins.^27^ In contrast, the NDI-BSA showed a peak with average *D*_h_ of 160 nm which is consistent with the particle size obtained from TEM (Fig. 1a) and thus confirmed nano-assembly of the bioconjugate SSDU–protein conjugate. On the other hand, co-assembled NDI-BSA + NDI-1 (3 : 7) showed no peak corresponding to the homo-assembly of NDI-BSA, instead a new peak with *D*_h_ close to 1.0 μm was noticed. While for an anisotropic structure like fibrils, the particle size obtained from DLS may not be accurate, the absence of any peak corresponding to the homo-assembly confirmed complete co-assembly of NDI-BSA and NDI-1 in their mixture.

Furthermore, nanoparticles of NDI-BSA showed a zeta potential value for −22 mV which was significantly higher than that of the free protein (−6.0 mV) which can be attributed to the aggregation of multiple proteins leading to a display of multiple negative charges on the surface of the resulting nanoparticles. However, a co-assembled fibre of NDI-BSA + NDI-1 (3 : 7) exhibited a smaller zeta potential value of −10 mV indicating relatively less surface charge density.

**Scheme 3:**
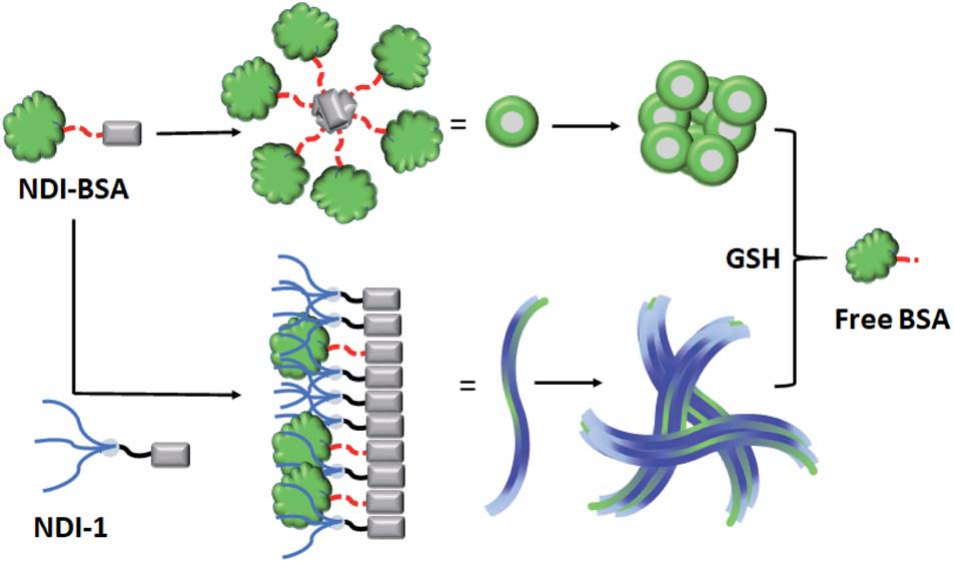
Proposed model for glutathione (GSH) responsive self-assembly of NDI-BSA and its co-assembly with NDI-1.

Based on these results it is proposed (Scheme 3) that NDI-BSA self-assembles by π-stacking among the NDI chromophore with rotational displacement along the long axis to accommodate the appended globular proteins, leading to the formation of small particles, which coalesce to produce larger nanoparticles, as seen in the TEM image (Fig. 1a). On the other hand, in the co-assembly of NDI-BSA and NDI-1, the two different building blocks when stack in a statistical fashion, the globular proteins are not likely to be located on top of each other which helps in avoiding the steric repulsion unlike the situation in the homo-assembly of NDI-BSA. Thus, probably the stacking mode of the SSDU in the case of the co-assembly is similar to that in the homo-assembly of NDI-1, which leads to the 1D growth producing fibres with the surface decorated with protein and the oligo-oxyethylene wedge. Lateral clustering among them leads to thicker bundles (as seen in the TEM image) with encapsulated proteins in the oligo-oxyethylene dominated hydrophilic interior domain.

**Fig. 3:**
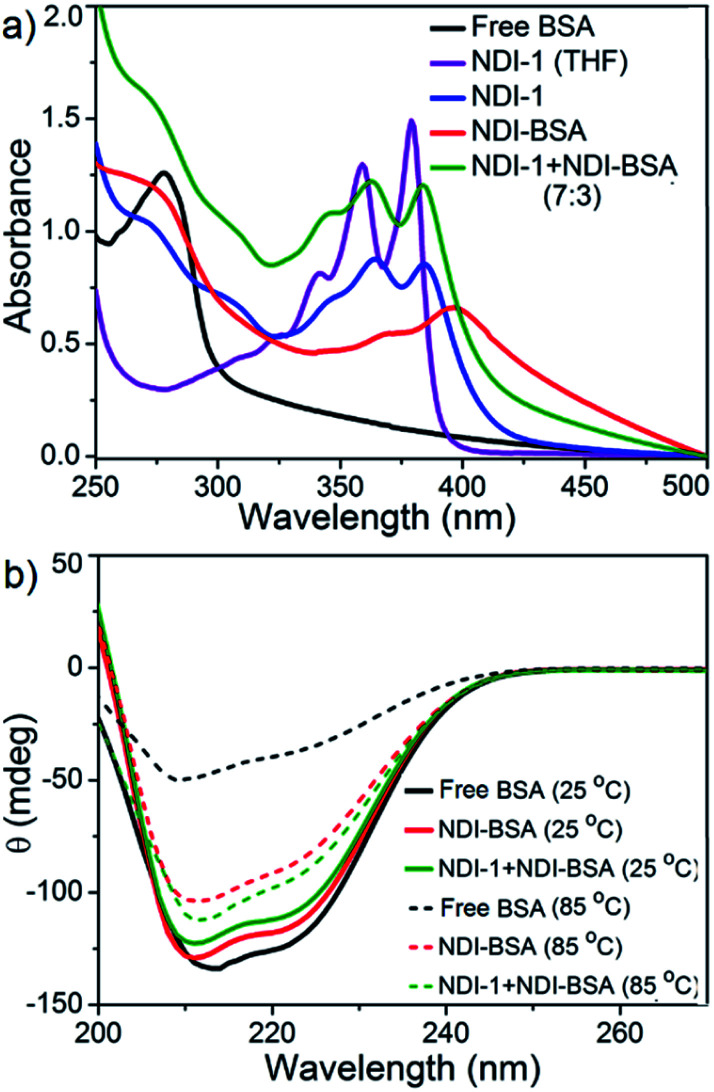
(a) UV/Vis spectra of BSA, NDI-1, NDI-BSA and NDI-1 + NDI-BSA (7 : 3) in water and comparison with the monomeric dye (NDI-1) in THF. *c* = 0.1 mM, *l* = 0.1 cm; (b) temperature dependent CD spectra of BSA, NDI-BSA and NDI-1 + NDI-BSA (7 : 3). *c* = 0.1 mM, *l* = 0.1 cm.

The UV/Vis spectra of NDI-BSA in water (Fig. 3) showed absorption bands in the window of 250–500 nm. A peak with *λ*_max_ = 270 nm can be attributed to BSA, and two other broad peaks with *λ*_max_ = 368 nm and 395 nm are assigned to the NDI chromophore. The UV/Vis spectrum of NDI-1 in THF showed sharp absorption bands with *λ*_max_ = 356 nm and 375 nm, indicating the presence of the monomer dye. A clear difference in the UV/Vis spectra of NDI-BSA in water (such reduced intensity, peak broadening and significant bathochromic shift of 20 nm), when compared to monomeric dye in THF indicated off-set π-stacking among the NDI chromophores^28^ in aqueous self-assembly of NDI-BSA. UV/Vis spectra of NDI-1 showed similar features but not identical to that of NDI-BSA. For example, the bathochromic shift was less prominent in this case. The UV/Vis spectrum of NDI-1 + NDI-BSA (7 : 3) also showed aggregated features but the spectrum was more similar to that of aggregated NDI-1 rather than NDI-BSA which corroborates with their similar morphology (Fig. 2). Based on these results, it is conceivable that the stacking mode of NDI-BSA is distinctly different than that in NDI-1 or NDI-1 + NDI-BSA (7 : 3). In the case of NDI-BSA, the presence of the globular protein probably inhibits face to face stacking and leads to off-set stacking with rotational displacement leading to spherical aggregates. However for NDI-1 or its supramolecular conjugate with NDI-BSA, less longitudinal displacement is evident from a relatively small bathochromic shift, which results in the formation of the elongated 1D structure (Scheme 3).

The UV/Vis spectrum of NDI-BSA at 85 °C showed that the features related to stacking of the NDI chromophores remained intact (Fig. S7†), indicating high thermal stability. Nevertheless, a sharp increase in the baseline intensity for NDI-1 or its co-assembly with NDI-BSA was noticed >40 °C (Fig. S8†) which is attributed to the lower critical solution temperature (LCST) due to the presence of the thermo-responsive oligo-oxyethylene chains of the hydrophilic wedge.^29^ CD spectra of NDI-BSA or its co-assembly with NDI-1 at 85 °C showed (Fig. 3b) almost identical features when compared to the spectra at room temperature, indicating that the thermal denaturation of the protein could be avoided by its supramolecular conjugation and assembly. In contrast, the CD spectrum of the native protein at high temperatures was significantly different than that at room temperature (Fig. 3b) which is attributed to the thermal denaturation of BSA as is reported at *T* > 70 °C.^30^

### Glutathione responsive bioactivity

**Fig. 4:**
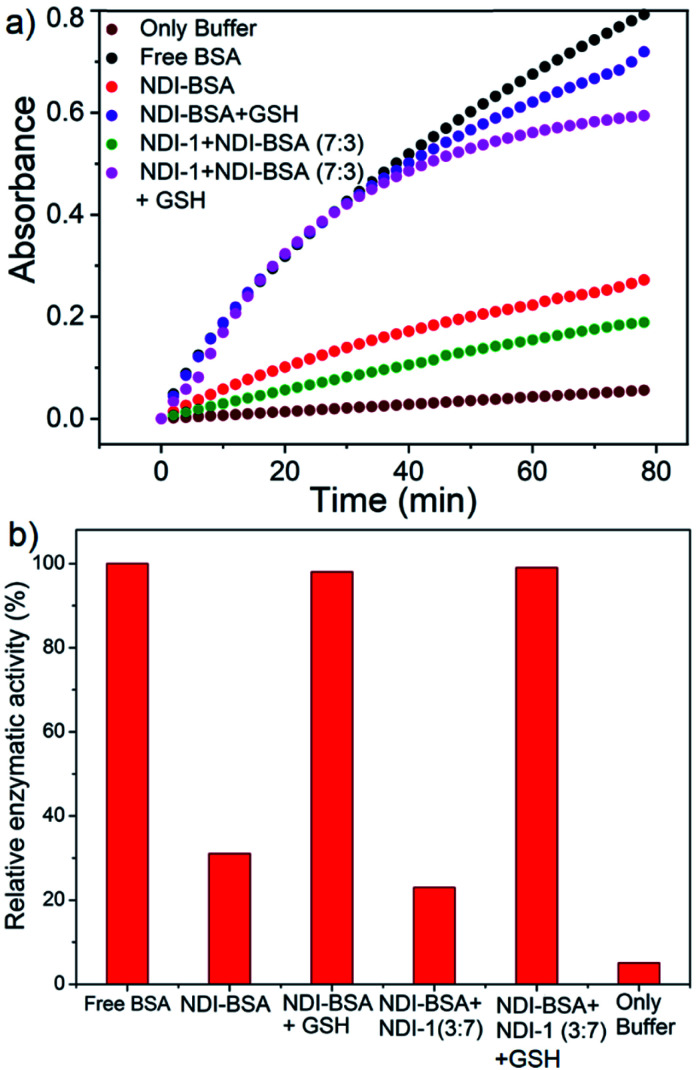
(a) Time dependent change in absorption of *p*-nitro phenol (*λ* = 405 nm) produced by hydrolysis of the NPA substrate with BSA in different forms as indicated in the figure. *T* = 25 °C; concentration of BSA and NPA = 0.1 mM and 10 mM, respectively; (b) relative enzymatic activity of BSA in different samples as estimated from the slope of each curve for the first 20 minutes and assuming free BSA activity as 100%.

BSA is known to exhibit esterase like activity^31^ against aryl esters such as 4-nitrophenyl acetate (NPA). This assay has been used to probe the conformational integrity of the protein in different BSA–polymer conjugates.^32,36*b*,*c*^ To examine that, solutions of free BSA, NDI-BSA and NDI-1 + NDI-BSA (7 : 3) were incubated with NPA in PBS buffer (pH-8) at 37 °C which resulted in production of *p*-nitro phenol as indicated by the appearance of a new peak in the UV/Vis absorbance spectra (Fig. S9†). By monitoring the absorbance at *λ*_max_ (405 nm) as a function of time, bioactivity of BSA was determined in its native state and self-assembled state (Fig. 4). Bioactivity of free BSA was set as 100% active and those of the other samples were estimated from the relative slope of the *p*-nitro phenol production curve over the first 20 minutes. A blank experiment, performed in only buffer without any BSA, indicated the negligible impact of the medium on the hydrolysis of NPA. It was noticed that in self-assembled NDI-BSA, the bioactivity of BSA was drastically reduced and estimated to be merely 31% in comparison to that of the free BSA. In the supramolecular conjugate of NDI-1 + NDI-BSA (7 : 3), it further decreased to 23%. It is noteworthy that the CD experiment provided clear evidence that the protein secondary structure was intact in the self-assembled state of NDI-BSA or its co-assembled state with NDI-1. Therefore, the observed activity inhibition may be related to inaccessibility of the active site by the substrate as the protein remained encapsulated in the core of the multi-particle aggregate in the case of NDI-BSA or bundles of fibres in the case of the co-assembly with NDI-1 (Scheme 3).

**Fig. 5:**
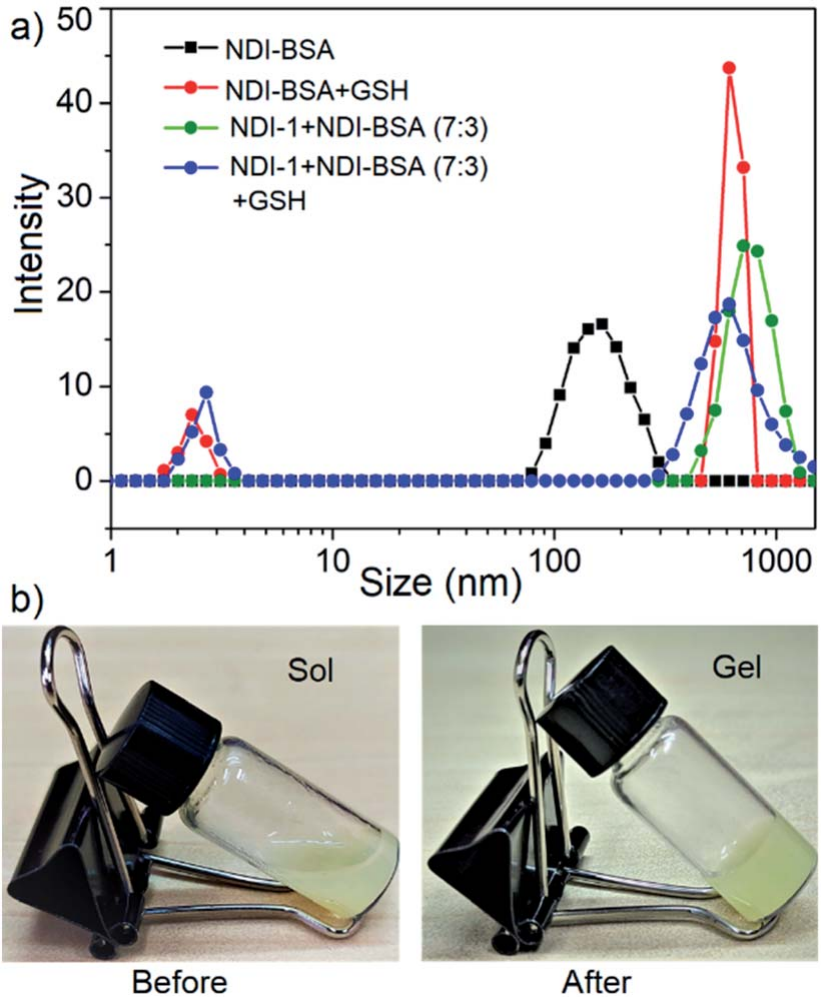
(a) DLS profile NDI-BSA and NDI-1 + NDI-BSA (7 : 3) before and after GSH (1.5 mM) treatment. *c* = 0.05 mM. Autocorrelation function corresponding to the DLS plots are shown in the ESI (Fig. S11†); (b) image of aqueous solution of NDI-1 + NDI-BSA (7 : 3) (*c* = 5.0 mM) before and after treatment with GSH (10.0 mM) and gentle heating.

In that case if the protein is released from the aggregate then it should regain its activity like the free BSA. It is well known that the disulfide bond can be cleaved under reducing conditions such as in the presence of a tri-peptide glutathione (GSH). In fact, it is well established that the intra-cellular GSH concentration is significantly higher compared to extra-cellular concentration^33^ which has motivated us to explore disulfide containing polymers^34^ and bioconjugates^35^ for redox responsive intra-cellular drug delivery. To test such a possibility, NDI-BSA solution was treated with GSH (*c* = 1.5 mM) and bioactivity was checked. Interestingly, with addition of GSH, the bioactivity of NDI-BSA increased from 31% to 98% (Fig. 4) indicating a strong impact of GSH. DLS data of the GSH treated sample showed the original peak (*D*_h_ = 160 nm) corresponding to nanoparticles of NDI-BSA disappeared completely and in turn two new peaks appeared with *D*_h_ = 3 nm and 620 nm (Fig. 5a), which are assigned to the free BSA and larger aggregated particles, respectively, due to the cleavage of the disulfide linkage by GSH. MALDI-TOF mass spectrometry of the GSH-treated NDI-BSA showed (Fig. S10†) a clear peak shift and the new peak exactly overlapped with that of the reduced BSA, confirming GSH triggered production of the free protein from its conjugate with the SSDU. We further examined the GSH effect on supramolecular bioconjugate of NDI-1 + NDI-BSA (7 : 3). Interestingly in this case, after addition of GSH and gentle heating, the viscous solution was converted to hydrogel (Fig. 5b) indicating detachment of the protein so that the system behaved more like that of NDI-1 in the absence of the protein. In this case also the release of the protein could be confirmed from the DLS data (Fig. 5a) showing the appearance of a new peak with *D*_h_ = 3 nm. Consequently, the bioactivity of the GSH treated sample increased (Fig. 4) to almost 100% from 23% confirming GSH-triggered release of the protein (Scheme 3).

### Enzymatic degradation

In previously reported examples of protein–polymer conjugates,^19^ including BSA–polymer conjugates^36^ or supramolecularly assembled proteins,^23,37^ the enzymatic activity was reported to be retained. In contrast, the present example shows 70–80% inhibition albeit an intact protein secondary structure, indicating the self-assembly provided an effective avenue for encapsulating the protein from the external environment and consequently the substrate had limited access to the active site of the protein. We envisaged this might be particularly useful to provide the much-desired stability of the protein against enzyme degradation^38^ which is a major concern in protein-based therapy. We investigated the stability of BSA in its supramolecularly assembled states against various gastrointestinal enzymes which generally degrade protein by hydrolysing the backbone amide bonds and produce fragmented amino acids. Solutions containing free BSA, NDI-BSA or NDI-1 + NDI-BSA (7 : 3) were subjected to hydrolysis by the treatment of trypsin and α-chymotrypsin enzymes at 37 °C for 2 h. The relative rate of hydrolysis was determined by the *o*-phthaldialdehyde (OPA) spectrophotometric assay.^39^ In this assay, α-amino acid units, released after the hydrolysis, react with OPA and β-mercaptoethanol to form an adduct with a distinct absorbance band in the window of 300–400 nm and therefore by monitoring the intensity of this absorption band one can estimate the extent of hydrolysis.

**Fig. 6:**
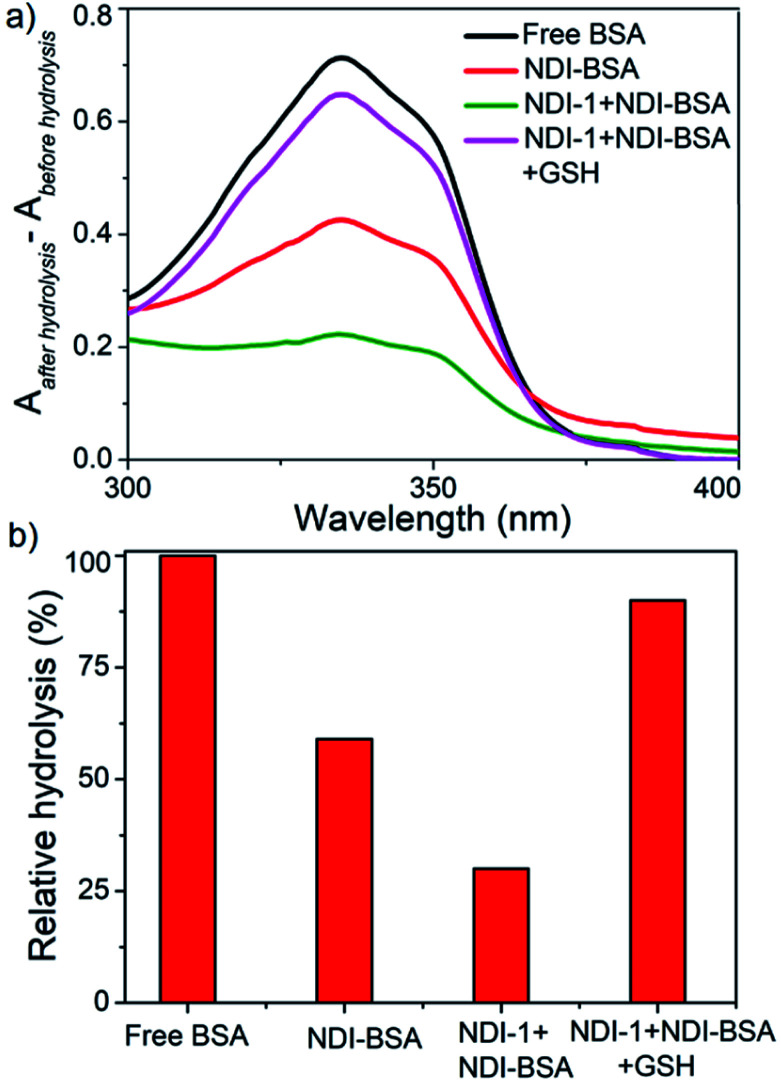
(a) Difference of UV-Vis spectra of various BSA samples treated with OPA before and after hydrolysis by enzymes (trypsin and α-chymotrypsin); (b) relative of hydrolysis of different samples.

However, in the present system, the NDI chromophore also absorbs in the same window and therefore the extent of hydrolysis in each case was estimated from the difference in absorbance of OPA-treated substrate with that of the untreated sample. The control experiment was performed under the same conditions but in the absence of any enzyme and substrate to ensure that the observed absorption change is exclusively related to the formation of the adduct. Data presented in Fig. 6 show a strong absorption band for free BSA indicating facile hydrolysis. It can be noticed that the band intensity for enzyme treated NDI-BSA or NDI-1 + NDI-BSA (7 : 3) were significantly less and by relative intensity of the band at *λ*_max_ (340 nm), the relative hydrolysis of BSA was determined to be 57% and 33% for NDI-BSA and its supramolecular conjugate with NDI-1, respectively, while the hydrolysis of free BSA was considered to be 100%. So, it is clear that the extent of hydrolysis of the protein has been substantially reduced by means of its supramolecular assembly governed by the attached SSDU and it further reduced when co-assembled with NDI-1. Interestingly, when the sample of NDI-1 + NDI-BSA (7 : 3) was pre-treated with GSH, the enzymatic hydrolysis was found to be >80%, further confirming that the encapsulation of the SSDU-attached protein in the interior of the bundles of the supramolecularly assembled nanostructures greatly helped in protection of the protein from enzymatic degradation.

**Fig. 7:**
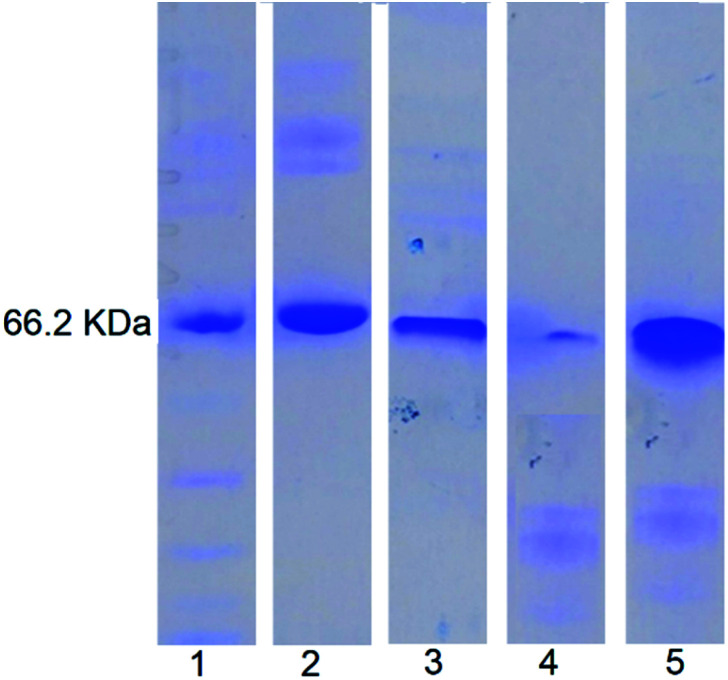
SDS-PAGE analysis: lane 1-MW markers; lane 2-BSA; lane 3-NDI-BSA; lane 4-enzyme treated BSA; lane 5: enzyme treated NDI-1 + NDI-BSA (7 : 3).

SDS-PAGE analysis (Fig. 7) showed that for the free BSA, after treating with enzymes for 2 h, the blue band completely disappeared indicating complete digestion. In contrast, no significant change in the blue band intensity was noticed for the enzyme treated NDI-1 + NDI-BSA (7 : 3) confirming that the protein mostly remained intact in this case. Therefore, it is evident that supramolecular conjugation and SSDU-regulated self-assembly could protect the protein from enzymatic hydrolysis and preserve the biological functions which should be of great relevance for protein delivery.

## Conclusions

We have demonstrated highly stable and stimuli-responsive supramolecular assembly of an engineered protein (BSA), attached with a supramolecular structure directing unit (SSDU) by a redox responsive disulfide bond. Specific molecular assembly among the SSDU leads to the formation of spherical nanoparticles (*D*_h_ = 150–200 nm). The same SSDU when attached with a small hydrophilic wedge instead of the protein, produces 1D fibres which could act as a host scaffold for impregnating the SSDU-attached protein, producing bundles of protein-appended fibres. Either in spherical nanoparticles or fibrillar co-assembly, the protein gained excellent thermal stability as its denaturation could be supressed and the protein secondary structure remained intact even at elevated temperatures. A significant inhibition was noticed in the esterase like activity of BSA in its self-assembled state, indicating inaccessibility of the substrate to the active site of the protein. In the presence of glutathione, due to the cleavage of the disulfide linker, the protein could be released resulting in regaining its bioactivity almost fully. Such supramolecular assembly protected the protein from enzymatic degradation to a great extent as the enzymatic hydrolysis reduced to approximately 30% for the co-assembled protein in comparison to the free protein. Although various protein–polymer conjugates have been reported in the literature with the objective to minimize the enzymatic degradation, we are not aware of a single report which elucidates such effects in reality. In contrast to protein–polymer conjugates, the present system is conceptually different as it demonstrates supramolecular polymerization/copolymerization of proteins and hierarchical assembly which clearly appears to have advantages with regard to rendering thermal and hydrolytic stability. Glutathione triggered release of the protein is particularly interesting and indicates the possibility of the protein remaining in the dormant state in the extracellular environment and released in active form in the intra-cellular environment or specific locations depending on the GSH concentration, which may have lasting impact in protein delivery. Finally, the present supramolecular assembly strategy is not limited to either the specific SSDU or protein, and thus considering the vast knowledge developed in the supramolecular polymerization of various π-systems^40^ and their rich photophysical properties, it is possible to explore similar approaches for wide-ranging functional supramolecular bioconjugation of other proteins and bio-molecules^41^ for theranostic applications.

## Conflicts of interest

There are no conflicts to declare.

## Supplementary Material

SC-012-D0SC05312K-s001
