# Supplementary material for: Redox responsive activity regulation in exceptionally stable supramolecular assembly and co-assembly of a protein
Source: Chem Sci. 2020 Nov 17;12(3):1101–8. doi: 10.1039/d0sc05312k (PMC8179030; doi:10.1039/d0sc05312k)
Supplement: SC-012-D0SC05312K-s001 [file SC-012-D0SC05312K-s001.pdf]

Electronic Supplementary Material (ESI) for Chemical Science.
This journal is © The Royal Society of Chemistry 2020

Supporting Information for

Redox Responsive Activity Regulation in Exceptionally Stable Supramolecular Assembly and Co-assembly of a Protein

Saptarshi Chakraborty, Rajesh Khamrui and Suhrit Ghosh*

School of Applied and Interdisciplinary Sciences, Indian Association for the Cultivation of Science, 2A and 2B Raja S. C. Mullick Road, Kolkata, India-700032

Materials and methods: All the reagents and solvents were purchased from commercial sources and purified by standard methods.[1] BSA (lyophilized powder) was purchased from Sigma Aldrich Chemical Co. HPLC grade solvents were used for all the spectroscopic studies and other experiments. ^{1}H-NMR were recorded on a Bruker DPX- 400 MHz or 500 MHz NMR spectrometer using TMS as standard. Mass spectra were taken on a Q-tof-micro quadruple mass spectrometer by electron spray ionization (ESI) technique. UV/Vis spectra were recorded at a Perkin Elmer Lambda 25 spectrometer. MALDI-TOF experiment was done in Ultraflextreme mass spectrometer (Bruker Daltonics) equipped with Bruker smartbeam II 355 nm Nitrogen laser using sinapinic acid as matrix. J-815 Circular Dichroism (CD) spectropolarimeter was used to carry out circular dichroism experiments. Dynamic Light Scattering (DLS) measurements were carried out in Malvern instrument at a scattering angle of 173°. TEM images were captured in JEOL-2010EX instrument operating at an accelerating voltage of 200 KV.

Synthesis and Characterization: Compound **1** and **2** were synthesized following literature reported procedure.[2-3]

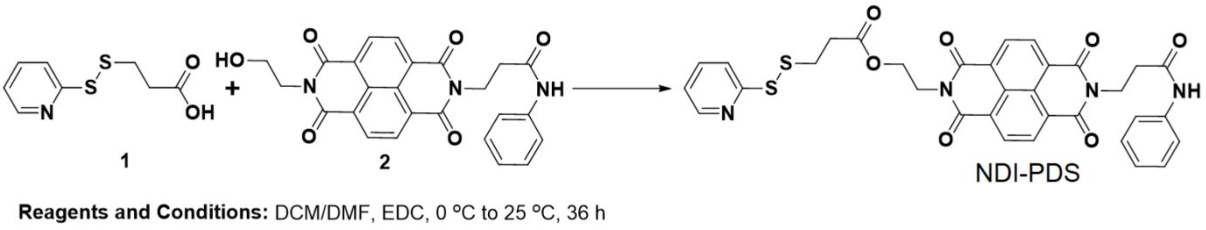

Scheme S1: Synthesis of NDI-PDS

NDI-PDS: Compound **1** (85 mg, 0.39 mmol) and **2** (180 mg, 0.39 mmol) were taken in a 25 mL round bottom flask and dissolved in a mixture of dry DCM (2 mL) and dry DMF (4 mL). To this solution, DMAP (10 mg, 0.2 mmol) was added and the mixture was stirred at 0 °C for 15min. Subsequently, a solution of 1-Ethyl-3-(3-dimethylaminopropyl) carbodiimide (90 mg,

0.58 mmol) in dry DCM (2 mL) was added drop wise to the reaction mixture and it was stirred at rt for 36 h under inert atmosphere. Then the stirring was stopped and the reaction mixture was washed with aqueous HCl (1N) solution (3 x 10 mL) followed by brine (1 x 10 mL) and dried over anhydrous Na_2SO_4. Then excess solvent was evaporated to get the crude product. It was further purified by column chromatography using silica gel as the stationary phase and 2% MeOH/DCM as the eluent to get the pure product as a light-yellow solid (NDI-PDS). Yield= 190 mg (75%); M.P. = 125 °C; ^{1}H NMR (500 MHz, $CDCl_3$, TMS): δ (ppm): 8.74 (s, 4H), 8.41 (d, 1H), 7.71(s, 1H), 7.61-7,58 (m, 2H), 7.50 (d, 2H), 7.29 (t, 2H, J = 7.5 MHz), 7.10 (m, 2H), 4.61 (t, 2H, J = 7 MHz), 4.52-4.48 (m, 4H), 2.97-2.89 (m, 4H), 2.70 (t, 2H, J = 7 MHz). ^{13}C NMR (100 MHz, $CDCl_3$): 168.5, 166.0, 163.0, 162.9, 159.7, 152.3, 149.4, 137.6, 142.5, 131.4, 131.3, 129.0, 127.0, 126.7, 126.6, 121.1, 120.5, 120.4, 58.7, 39.7, 37.3, 35.0, 34.02, 33.12.

Reduction of BSA and quantification of free thiols using Ellman's assay: BSA was reduced with dithiothreitol (DDT) using a literature reported procedure.[4] Native BSA (100 mg) was dissolved in 0.1M PBS buffer (pH=8.3) solution, containing 1.0 mM ethylenediaminetetraacetic acid (EDTA). Solution was degassed for 20 min by purging Ar gas. DDT was dissolved in PBS buffer (1 mg in 200 µl) and added dropwise to BSA solution which was stirred for 4h at rt. After that, the solution was dialyzed for 48 h using deionized water (3500 M.W cut off membrane) and lyophilized to obtain reduced BSA as white powder. Quantification of free thiol was performed via Ellman's assay (Fig. S1a). Ellman's solution was prepared by dissolving 5-5□-Dithiobis (2-nitrobenzoic acid) (Ellman's reagent, 4 mg) in 0.1 M PB (pH 6.8, containing 2 mM EDTA). 5.0 mg of reduced BSA was dissolved in 500 µL of same buffer solution and to this Ellman's reagent (10 µL) was added and incubated for 45 minutes at ambient temperature. The absorbance (λ_{max} = 412 nm) of the 5-thio-2-nitrobenzoic acid was measured by UV-Vis spectroscopy. In an independent experiment, a standard calibration curve was constructed using 2-mercaptoethanol as the reference compound.[5] Firstly, various known concentrations of 2-mercapethaol solutions in 0.1 M PB (pH 6.8, containing 2 mM EDTA) were prepared and the same volume of Ellman's reagent (10 µL) was added to each vial. Then, all the solutions were incubated for 45 minutes at room temperature and the absorbance spectra were recorded (Fig. S1b) which showed evolution of a characteristic absorption band (λ_{max}: 412 nm) due to the production of the 5-thio-2-nitrobenzoic acid (TNB). Absorbance (at 412 nm) was plotted against the concentration of 2-mercaptoethanol (Fig. S1c) to get the calibration curve. Appending the

absorbance (at 412 nm) of the Ellman's reagent treated reduced BSA into this standard calibration curve, the free thiol concentration of the reduced BSA solution was estimated to be 0.144 mM which closely matched with the theoretical concentration (0.15 mM) and thus indicated the free thiol content of 96 % in the reduced BSA sample.

Synthesis of NDI-BSA: To a degassed solution of reduced BSA (100 mg) in PBS buffer (5.0 mL; pH 6.8) containing 2.0 mM EDTA and 150 mM NaCl, a solution of NDI-PDS (19.6 mg, 20 equivalent) in DMF (0.5 mL) was added dropwise which resulted in yellow coloration. The reaction mixture was allowed to stir at room temperature (25-30 °C) for 12 h and occasionally the absorption (λ=390 nm) was checked to monitor the progress of the reaction. Subsequently, the solution was transferred to a dialysis bag (MW cut-off: 7000) and dialysis was continued for 24 h against 9.5:0.5 water/DMF mixture and then with deionised water for another 24 h to remove excess reactant (NDI-PDS) and water-soluble by-products. Then the solution was freeze-dried to obtain the desired product as a yellowish solid powder.

MALDI-TOF experiment: Matrix assisted laser desorption ionization time-of-flight (MALDI-TOF) mass spectrometry was performed on a Bruker Daltonics Flex-PC machine. Mass spectral measurement were done in positive reflectron mode using Sinapinic acid as a matrix. Native BSA, reduced BSA, NDI-BSA or GSH treated NDI-BSA (c = 1.0 mg/mL) was mixed with matrix in 1:1 ratio (v/v). 2.0 μL of this matrix-compound mixture was deposited on the wells of a 384 well ground-steel plate, dried on the spot and spectra were recorded.

Sample preparation: Measured amount of NDI-BSA or NDI-1 or their mixture (3: 7) was taken and directly dissolved in water or buffer by gentle heating and sonication to make a clear solution with concentration required as per a particular experiment. This solution was allowed to equilibrate for 1h before doing any physical studies.

UV/ Vis or CD spectroscopy studies: CD measurements in the far-UV region were carried out at 25 °C or 85 °C for dilute solutions (c = 0.1 mM; buffer, pH 8.1) of NDI-BSA or its mixture with NDI-1 in the wavelength range of 280-190 nm using a cuvette of path length of 1.0 mm, scan speed 50 nm/min, and a response time of 2.0 s. The spectra were averaged over five scans to minimize noise. Same samples were used for UV/Vis spectroscopy studies with (l=0.1cm).

Molecular weight estimation of NDI-BSA by UV/Vis spectroscopy method: Extinction coefficient of NDI-1 in DMF was estimated by concentration dependent UV/Vis spectroscopy (Fig. S2). On the other hand, a solution of NDI-BSA in water (2.0 mg/mL) was

treated with glutathione (10 mg) and stirred at rt for 24 h. The mixture was freeze dried and the resulting powder was dispersed in DMF (1.0 mL), centrifuged to separate the insoluble part (protein), the clear supernatant was collected and analysed by UV/Vis spectroscopy (Fig. 1b). Correlating the band intensity at λ = 379 nm and the extinction coefficient estimated for NDI-1 (Fig. S2), the concentration of NDI chromophore could be calculated and from this value the molecular weight of NDI-BSA was estimated.

Microscopy (TEM and AFM) studies: A solution of NDI-BSA or NDI-1 or their mixture in water (*c* =0.05 mg/mL) was prepared and 10 μL of the solution was drop-casted on a carbon-coated copper grid and blotted with Whatman 50 filter paper and allowed to air-dry for 24 h before capturing TEM images. The same solution was spin-coated on Mica and allowed to air-dry for 24 h before capturing AFM images.

Cell culture condition and cytotoxicity assay: Human cervical cancer cell line (HeLa) was cultured in a high glucose Dulbecco's Modified Eagle Medium (DMEM) containing 10% fetal bovine serum and 1% L-Glutamine-penicillin-streptomycin at 37 °C in a humidified environment containing 5% CO_2. Cells were maintained by passaging them regularly at ~ 80% confluency. Metabolic activity of HeLa cells in presence of NDI-BSA and BSA was probed using 3-(4, 5-dimethylthiazol-2-yl)-2, 5-diphenyltetrazolium bromide (MTT) assay. Cells were seeded in 96-well plates with a seeding density of 10,000 cells/ well and after 24 h the medium was replaced by BSA or NDI-BSA solutions (*c* = 0-500 μg/mL) and then cells were then incubated for 12 h, 24 h and 48 h. Subsequently 50.0 μL of freshly prepared MTT (2.5 mg/mL in DMEM) solution was added into each well. The medium was removed after 4 h incubation at 37 °C and 100.0 μL DMSO was added into each well and the plates were gently shaken for 10 min at rt to dissolve all precipitates formed. The absorbance of MTT at 570 nm was monitored by a microplate reader (VARIOSKAN, Thermo Fisher). Cell viability was expressed by the ratio of the absolute absorbance of the cells incubated with BSA or NDI-BSA. These experiments were repeated three times independently with three replicates each time and data are presented as mean ± SD, n = 3.

Bioactivity assay of BSA: In a typical experiment, a solution (*c*= 0.1 mM, volume- 200 μL) of NDI-BSA or NDI-BSA + NDI-1 (3: 7) was prepared in PBS buffer (pH-8). To this solution, 10 μL solution of the substrate NPA in acetonitrile (10 mM) was added and the homogeneous mixture was transferred to a quartz cuvette placed in the UV/Vis spectrophotometer at 25 °C. The hydrolysis of NPA was monitored by measuring the absorbance of the 4-nitro-phenolate anion at 405 nm for 80 minutes. A control experiment

was performed under identical condition using the free BSA and its activity was considered as 100 %. For studying the impact of glutathione (GSH) triggered release of the protein on its bioactivity, GSH (*c*= 1.5 mM) was added to aqueous solution of NDI-BSA or NDI-BSA + NDI-1 (3: 7) (*c*= 0.1 mM) and the mixture was stirred at room temperature under argon atmosphere for 24 h and subsequently the activity assay was performed following the procedure described above. Effect of only GSH on hydrolysis of the substrate was checked independently and its effect was subtracted from the actual data.

Enzyme hydrolysis experiment: Solution (2.0 mg/mL) of BSA or NDI-BSA or NDI-1+NDI-BSA (7:3) (Glutathione treated or untreated), was prepared separately in 50 mM sodium phosphate buffer (pH= 8). To the solution of 450 µl of substrate, 25 µL (2.5 mg/500 µL) of trypsin from porcine pancreas and 25 µL (2.5 mg/500 µL) of α-chymotrypsin from bovine pancreas were added together and incubated at 37 °C for 2 h. After incubation, all samples were heated at 90 °C for 5 min to stop the reaction. Hydrolyzed material was stored at -20 °C before doing the experiment. The *o*-phthaldialdehyde (OPA) solution was prepared by combining 25.0 mL of 100 mM sodium tetraborate, 2.5 mL 20% SDS, 40.0 mg of OPA in 1.0 mL methanol and 100 µL of β-mercaptoethanol and 21.4 mL water to a total volume of 50 mL. To assay proteolysis, 50 µL of the hydrolysates was added to 450 µL of the OPA solution, mixed well and incubated for 2h in at room temperature. Then the absorbance at 340 nm was measured in a spectrophotometer. Absorbance of OPA solution itself (with any substrate) also measured and eliminated from samples' absorbance. Percentage of relative hydrolysis was expressed as the percentage of absorbance difference of substrate after and before hydrolysis by enzymatic digest.

Sodium dodecyl sulfate polyacrylamide gel electrophoresis (SDS-PAGE): SDS-PAGE experiment was carried out using a premixed electrophoresis buffer containing 25 mM Tris, 192 mM glycine and 0.1% SDS (Tris/Glycine/SDS buffer) to check the stability of BSA (in various assembled forms) against enzymatic degradation. All the samples were mixed with Laemmli buffer (1:1 v/v) solution and kept for 1h. The samples were resolved through a 10% polyacrylamide gel which was performed using a BioRad Mini-Protean II electrophoresis setup at a constant voltage (20 mA). After complete running, the gel was stained with 0.1 % (w/v) Coomassie brilliant blue and left for 2h. Stained gel was de-stained using a de-staining solution (25% Methanol+7% acetic acid+water) with continuous shaking until a typical blue band for protein samples were clearly visible. A molecular weight standard protein (Lane Marker) and the native BSA were used as references.

Additional Figures

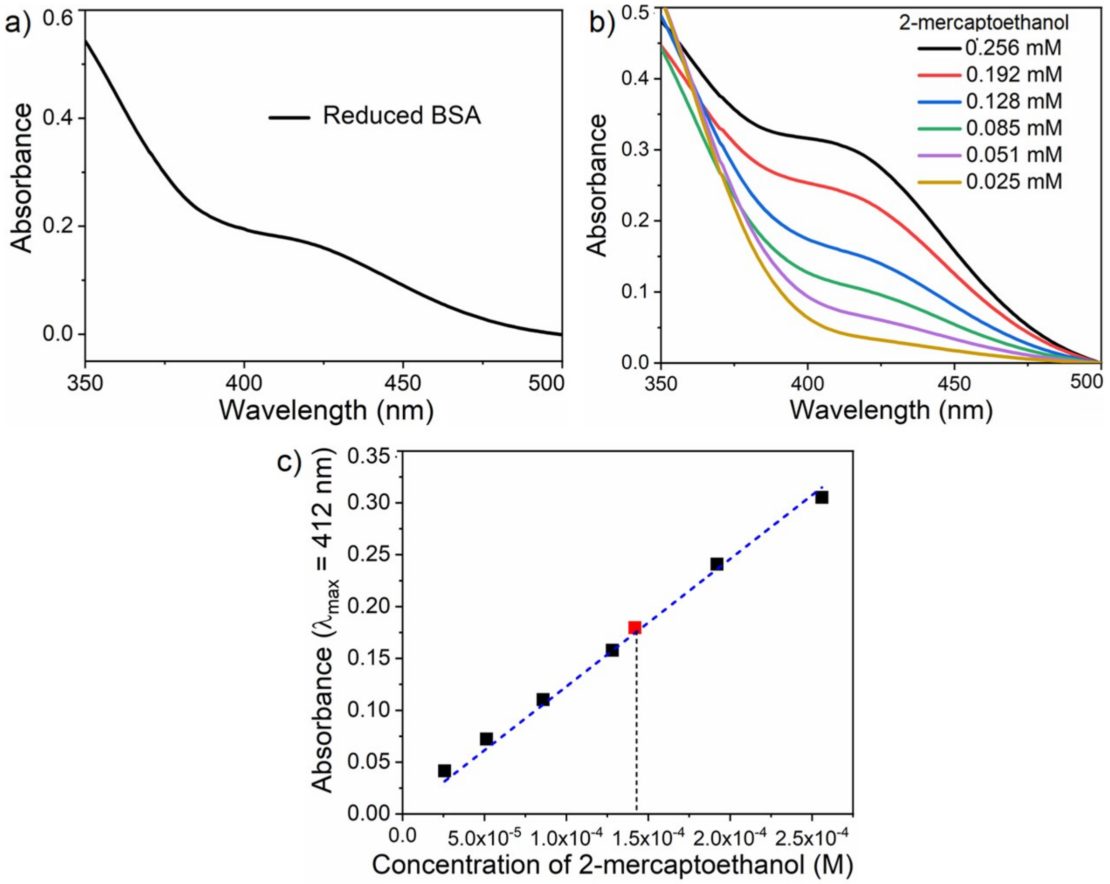

Fig. S1 a) UV-Vis spectrum of Ellman's reagent treated with reduced BSA (*c* = 5 mg/500 µL, *l* = 0.1 cm), b) UV-Vis spectra of Ellman's reagent treated with different solutions of 2-mercaptoethanol with varying concentration (*l* = 0.1 cm); c) Plot of absorbance (λ = 412 nm) as a function of 2-mercaptoethanol concentration; By feeding the absorbance at 412 nm (indicated by red square) from the spectrum shown in Fig. S1a (Ellman's reagent treated reduced BSA), the free thiol concentration in the reduced BSA sample was estimated to be 0.144 mM.

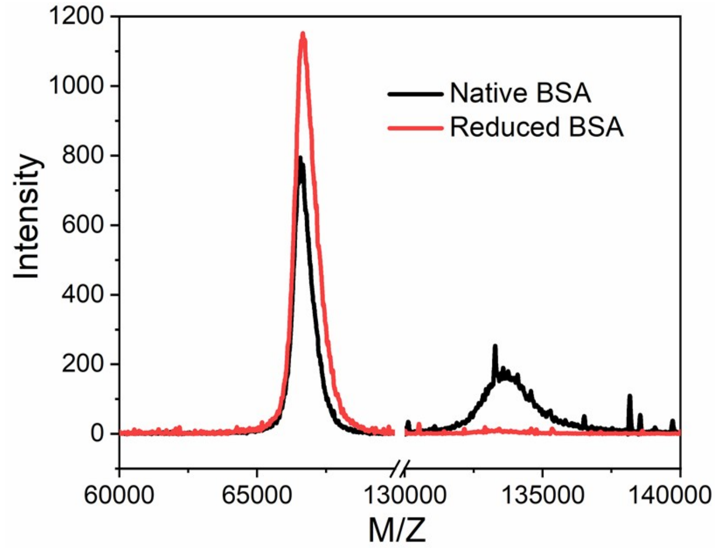

Fig. S2 MALDI-TOF MS spectra of native BSA and reduced; Matrix-Sinapinic acid. The dimer peak with M/Z of 133000 was absent in the reduced BSA..

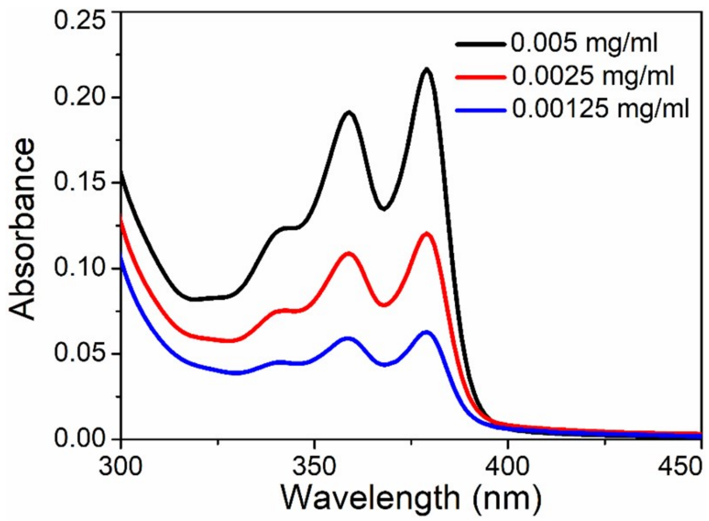

Fig. S3: Concentration dependent UV-Vis spectra of NDI-1 in DMF (l = 0.1 cm, T = 25°C). Extinction co-efficient at λ = 379 nm was estimated as 79.97 mL.mg^{-1}.cm^{-1}

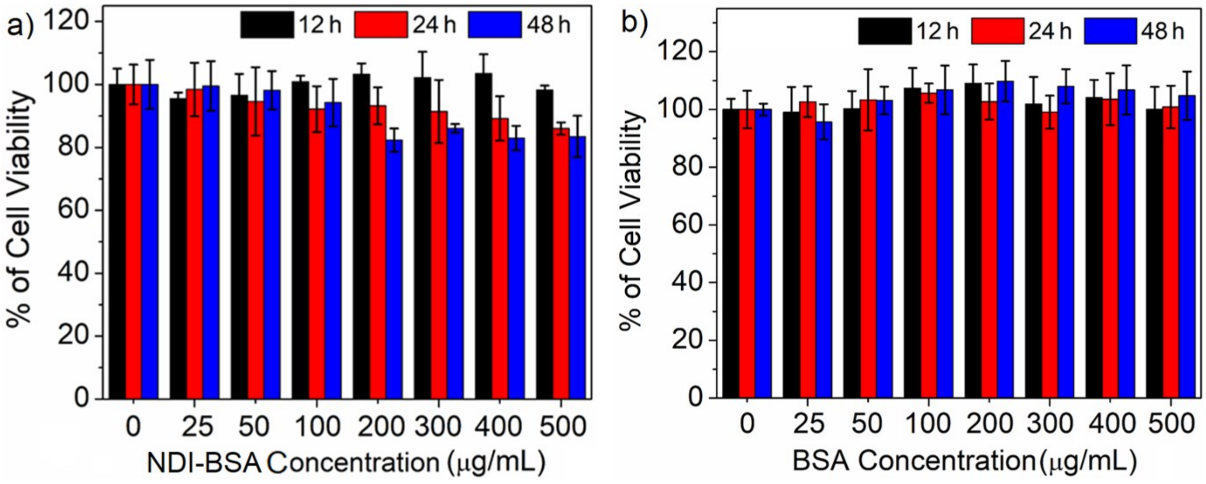

Fig. S4 Cell viability results for HeLa cells treated with a) NDI-BSA, b) BSA (c = 0-500 μg/mL) assessed by an MTT assay. Experiments were repeated three times independently with three replicates each time. Data are presented as mean ± SD.

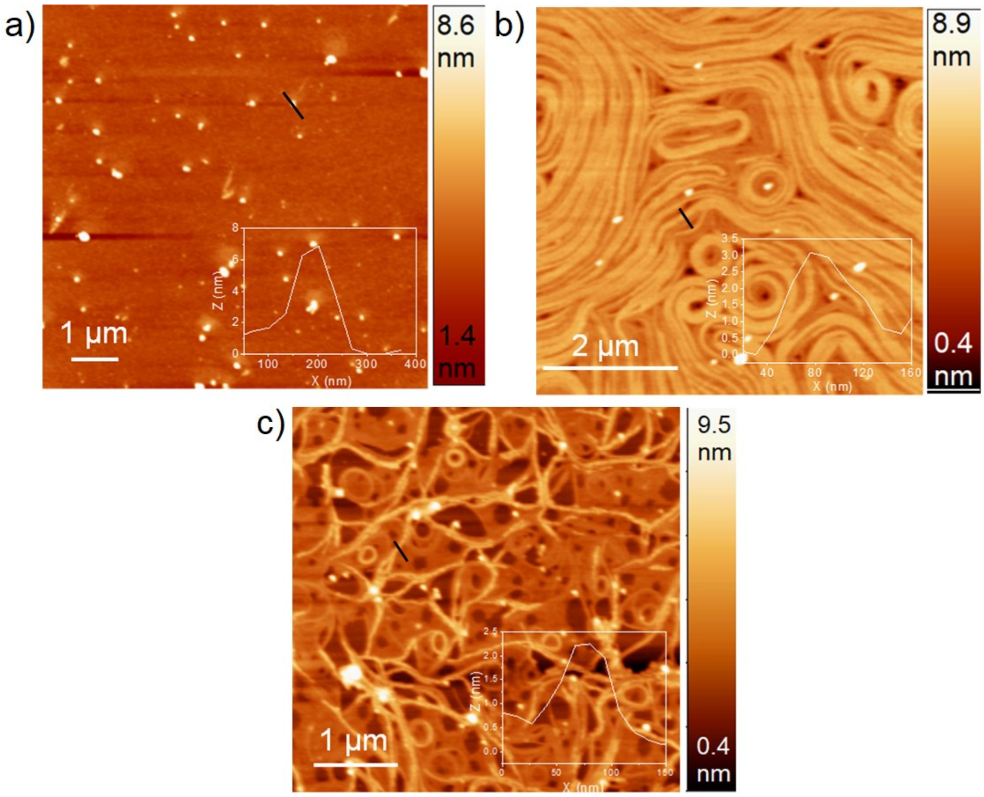

Fig. S5 AFM image of a) NDI-BSA, b) NDI-1 and c) NDI-1+NDI-BSA (7:3) samples prepared from the aqueous dispersion (c = 0.05 mM). Insets show the height profile along the black line. The images have been processed with software WS x M 5.0 Develop 8.4.

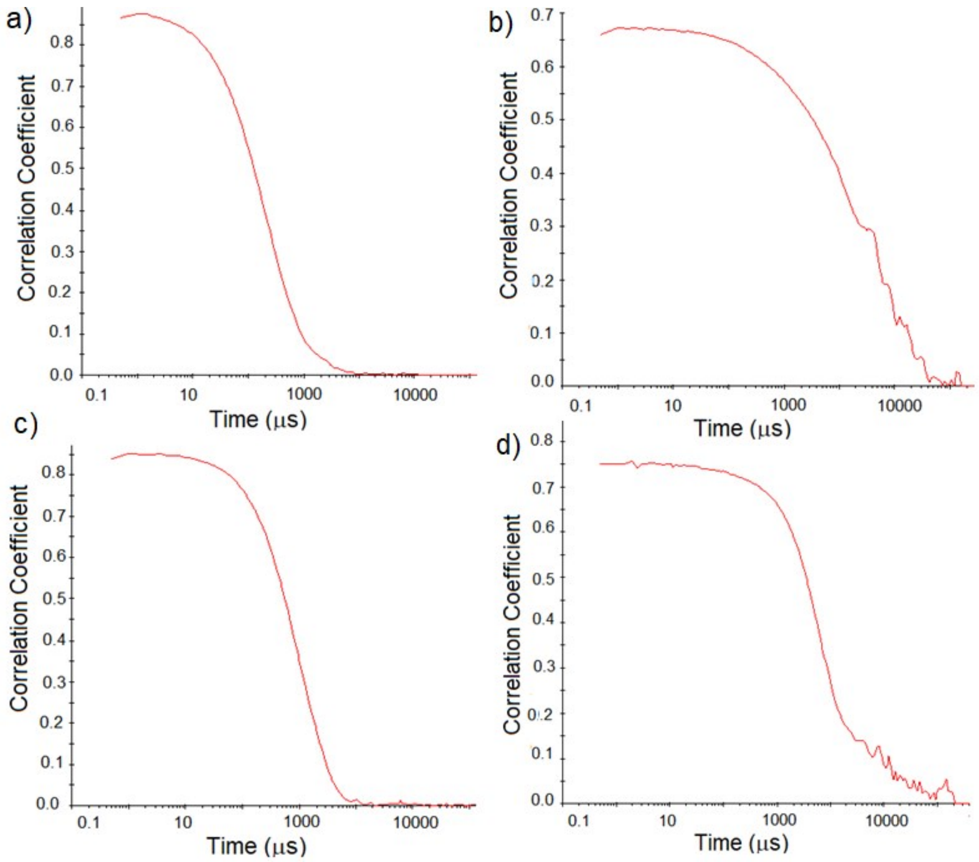

Fig. S6 Autocorrelation function corresponding to the DLS plots (shown in Fig, 2d) of a) Free BSA, b) NDI-1, c) NDI-BSA and d) NDI-1+NDI-BSA (7:3).

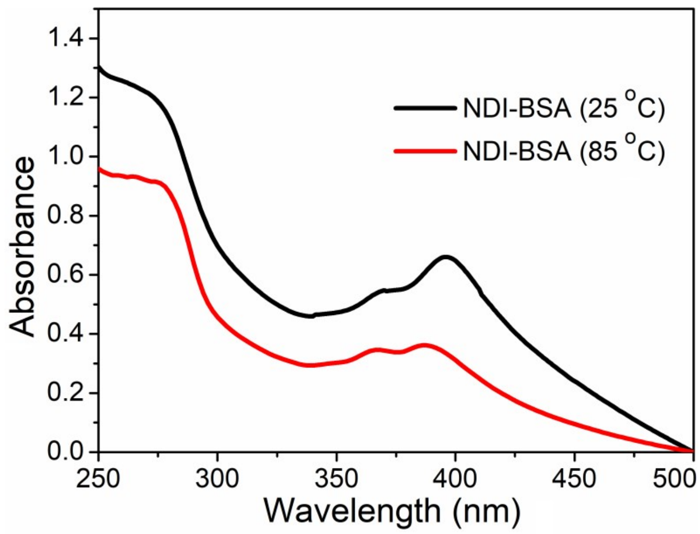

Fig. S7 UV-Vis spectra of NDI-BSA at 25°C and 85 ˚C; c = 0.1 mM, l = 0.1 cm.

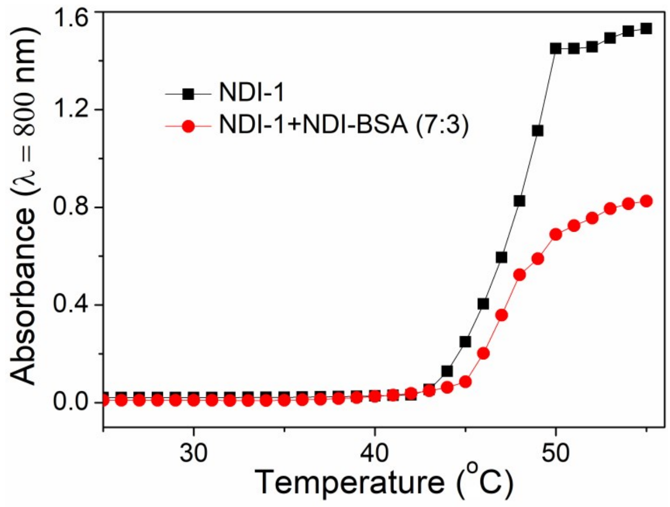

Fig. S8 Temperature dependent absorbance (monitored at 800 nm) of aqueous solution of NDI-1 and NDI-1+NDI-BSA (7:3); c = 0.1 mM in both samples.

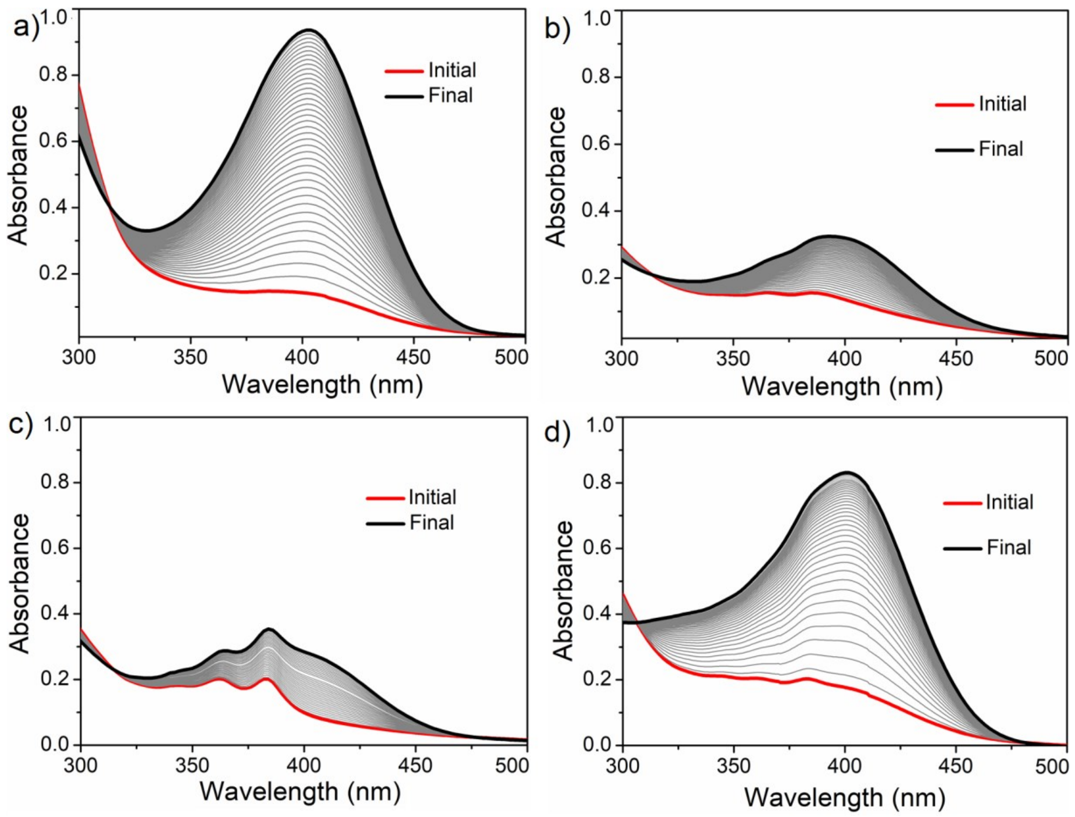

Fig. S9: Time dependent (Initial-0 min, Final-80 min) UV-Vis spectra of p-nitrophenol, generated after hydrolysis of substrate (NPA) by different forms of BSA: a) Free BSA, b) NDI-BSA, c) NDI-1+NDI-BSA (7:3) and d) NDI-1+NDI-BSA (7:3) + GSH for bioactivity assay, c = 0.1 mM for all cases.

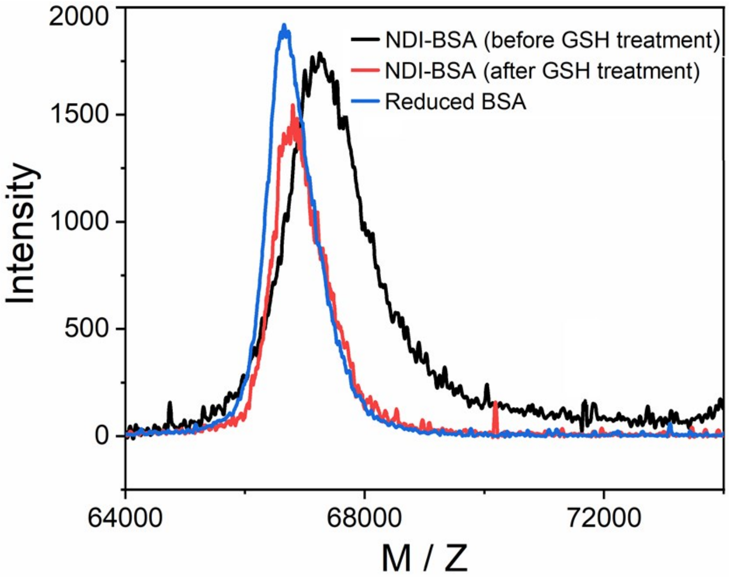

Fig. S10 Comparison of the MALDI-TOF MS spectra of NDI-BSA, before and after GSH-treatment; Matrix-Sinapinic acid. Clear peak shift after GSH treatment and overlap of the new peak with that of the reduced BSA confirm GSH triggered release of BSA from the NDI-BSA.

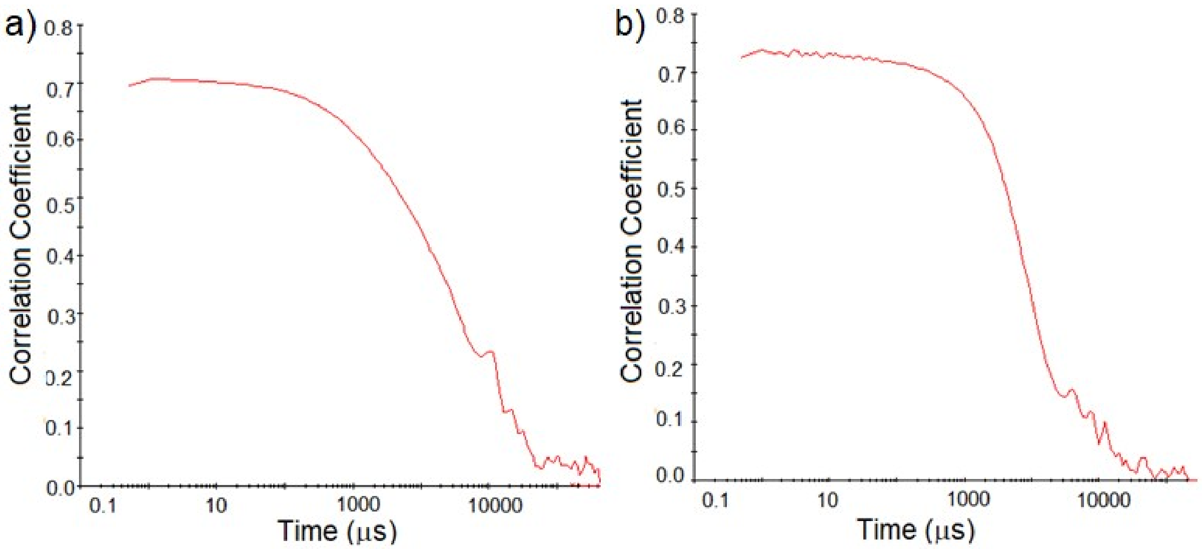

Fig. S11 Autocorrelation function corresponding to the DLS plots (shown in Fig. 5a) of NDI-BSA+GSH and b) NDI-1+NDI-BSA (7:3) +GSH.

Reference:

1 D. D. Perrin, W. L. F. Armarego, D. R. Perrin, *Purification of Laboratory Chemicals*, 2nd ed., Pergamon, Oxford, 1980.

2 A. Sikder, D. S. Ray, V. K. Aswal and S. Ghosh, *Chem. Eur. J.,* 2019, **25**, 10464-10471.

3 P. Rajdev, S. Chakraborty, M. Schmutz, P. Mésini and S. Ghosh, *Langmuir,* 2017, **33**, 4789-4795.

4 Z. Liu, C. Dong, X. Wang, H. Wang, W. Li, J. Tan, J. Chang, *ACS Appl. Mater. Interfaces.,* 2014, **6***,* 2393-2400.

5 J. F. Janatova, J. K. Fuller and M. J. Hunter, *J. Biol. Chem.,* 1968, **243**, 3612-3622.
